# Hospital Representation on the King's Fund

**Published:** 1921-10

**Authors:** 


### CORRESPONDENCE.

[Correspondence on all subjects is invited, but we cannot in any way be responsible for the opinions expressed by our correspondents, who must give their name and address as a guarantee of good faith, but not necessarily for publication. Correspondents are reminded that brevity of style and conciseness of statement greatly facilitate early insertion.]

### Hospital Representation on the King's Fund.

*To the Editor of* THE HOSPITAL AND HEALTH REVIEW.

SIR,—A straw indicates the flow of the stream, and the multiplicity of forms and circulars from the King's Fund have a significance which has not, I imagine, been lost upon hospital secretaries. If there is any ambiguity in the above remark it will speedily be removed by referring to the latter part of paragraph 15 of K.F.P. 45. Is it not high time that hospitals had full representation upon the Committee of the King's Fund?—Yours truly,

SECRETARY.

